# Pathway Reconstruction of Airway Remodeling in Chronic Lung Diseases: A Systems Biology Approach

**DOI:** 10.1371/journal.pone.0100094

**Published:** 2014-06-30

**Authors:** Ali Najafi, Ali Masoudi-Nejad, Mostafa Ghanei, Mohamad-Reza Nourani, Ali Moeini

**Affiliations:** 1 Laboratory of Systems Biology and Bioinformatics (LBB), Institute of Biochemistry and Biophysics, University of Tehran, Tehran, Iran; 2 Genomics Division, Chemical Injury Research Center, Baqiyatallah University of Medical Sciences, Tehran, Iran; 3 Department of Algorithms and Computation, College of Engineering, University of Tehran, Tehran, Iran; Mayo Clinic College of Medicine, United States of America

## Abstract

Airway remodeling is a pathophysiologic process at the clinical, cellular, and molecular level relating to chronic obstructive airway diseases such as chronic obstructive pulmonary disease (COPD), asthma and mustard lung. These diseases are associated with the dysregulation of multiple molecular pathways in the airway cells. Little progress has so far been made in discovering the molecular causes of complex disease in a holistic systems manner. Therefore, pathway and network reconstruction is an essential part of a systems biology approach to solve this challenging problem. In this paper, multiple data sources were used to construct the molecular process of airway remodeling pathway in mustard lung as a model of airway disease. We first compiled a master list of genes that change with airway remodeling in the mustard lung disease and then reconstructed the pathway by generating and merging the protein-protein interaction and the gene regulatory networks. Experimental observations and literature mining were used to identify and validate the master list. The outcome of this paper can provide valuable information about closely related chronic obstructive airway diseases which are of great importance for biologists and their future research. Reconstructing the airway remodeling interactome provides a starting point and reference for the future experimental study of mustard lung, and further analysis and development of these maps will be critical to understanding airway diseases in patients.

## Introduction

Airway remodeling is a term used to describe the dynamic processes in obstructive airway diseases. It usually refers to epithelial layer injury followed by structural changes in the airways and lung architecture [1]. However, the cellular and molecular processes depend on the type and the state of disease and the patient. Consequences of airway remodeling could include a decrease in pulmonary function and reduced responsiveness to bronchodilator therapy. Airway remodeling is reported in complex diseases such as asthma, chronic obstructive pulmonary disease (COPD), and Mustard Lung as the main respiratory clinical sign. Also, progressive dyspnea and airflow limitations, mucostasis and mucosal inflammatory reaction are usually associated with airway remodeling [2], [3]. Mustard lung has an irreversible pattern of airway obstruction like COPD [4] without any evidence of emphysema. It is resistant to anti-asthma therapy and an irreversible pattern of obstruction. Based on these similarities with asthma and COPD, mustard lung can also be a good model for evaluation of airway remodeling.

There is a need for a holistic approach to decode the massive amount of data generated with modern biological approaches. Systems biology can integrate multilevel views of cell physiology data generated by low and high-throughput techniques into a comprehensive understanding of nonlinear molecular properties. Generation of high-throughput omics data, including genomics, proteomics and metabolomics enable us to simultaneously measure and analyze cellular components at any given condition. Currently, large databases of heterogeneous biological data are available including gene expression profiles (microarray, EST, and SAGE), interaction data, and catalogs of gene or protein functions. Also, many computational tools and algorithms have been developed to identify biological modules or pathways in the context of biological molecular networks [5]. Consequently, the systems biology strategy may be able to identify and construct novel pathways, and as such, is an emerging biological tool of great interest [6].

Although individual components of this molecular interaction data have been studied for decades, the accumulation of huge datasets to create molecular networks is a topical advance in the field of molecular medicine [7], [8]. Moreover, recent progresses in molecular biology have highlighted the necessity of a systems biology approach. So, reconstruction and disruption of biological networks and pathways, including metabolic pathways, protein-protein interaction networks (PPI), signal transduction pathways, and gene regulatory networks (GRN), has been a valuable tool in the abstraction of biological concepts [8]. Most studies in this field have focused on the reconstruction, analysis and modeling of intracellular and extracellular networks [9]. This approach becomes more important when applied to polygenic diseases for complex etiologies [10], [11], while disease or abnormal pathways such as airway remodeling are given less consideration. Analysis of disease pathways has the potential to elucidate the molecular mechanisms underlying disease progression and response to treatment. Accordingly, novel genes, proteins and pathways are reported in complex diseases such as cancer [12], Alzheimer disease [11], atherosclerosis [13], and Parkinson's disease [14], and these can be understood by utilizing PPI network models combined with gene expression data.

In this study, we attempt to describe the process of airway remodeling pathway in mustard lung [15]. Interestingly, more than 45,000 of 100,000 Iranian exposed patients are suffering from the late effects of sulfur mustard (SM; 2-bis-chloroethyl-sulfide) after almost 25 years post-exposure [4]. The chemical warfare agent sulfur mustard as a potent alkylating agent is highly reactive vesicant that can cause airway epithelial injury. Recent studies on Iranians of about 20–25 years in age after exposure to SM have shown the most common late complications in descending order of frequency are found in the lungs, eyes, and skin [4]. Damage to the epithelium layer is known as a key factor driving airway remodeling. Airway remodeling is the greatest cause of long-term disability among patients with combat-exposure to SM gas [16]–[18]. COPD and mustard lung are similar in clinical symptoms and signs, but differ at the molecular level and interactions between them.

**Figure 1:**
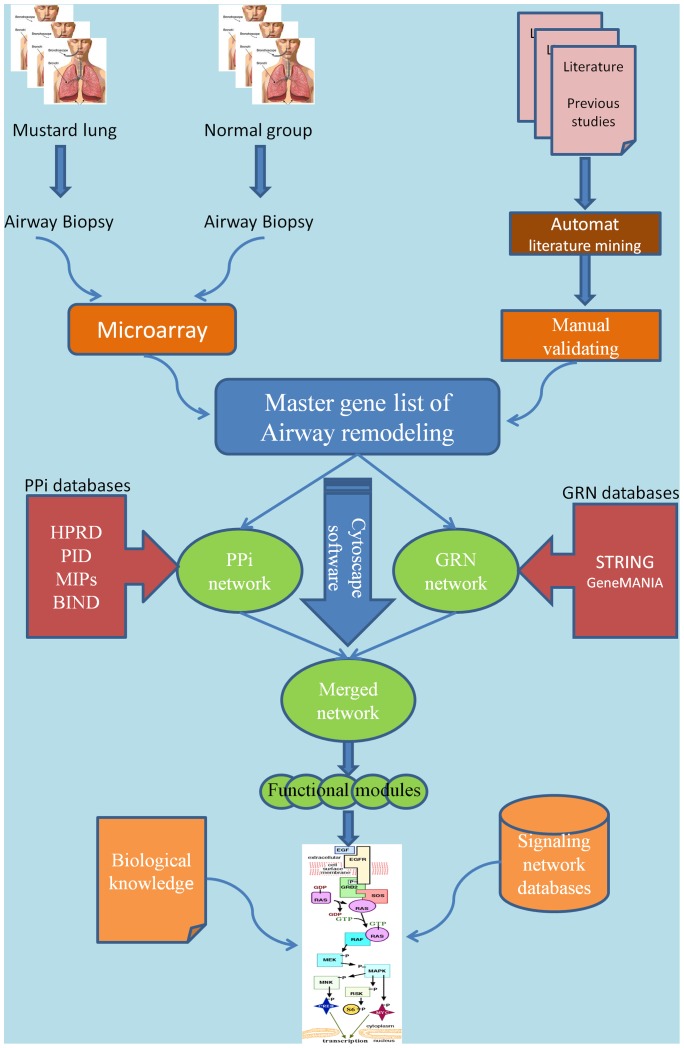
A schematic picture demonstrating the pathway reconstruction workflow. The workflow shows that the master gene list is provided from two sources (microarray gene expression and literature mining). Protein-Protein interaction network (PPi) and Gene Regulatory Network (GRN) are compiled using Cytoscape software and its plugins. Also, signaling pathway reconstruction is accomplished in CellDesigner software based on biological knowledge accumulated in related literatures and public databases (e.g. Gene Ontology, Uniprot, and NCBI geneRIFs) and related signaling pathways deposited in the signaling pathway databases such as Biocarta, Reactome, and NCI-PID. The final mustard signaling pathway was drawn manually using CellDesigner.

Accordingly, we have prepared a master list of mustard lung related genes. PPI and GRN networks were then generated and topological analysis was performed on merged network. Then, key signaling paths in networks were identified and mapped manually. The first reconstruction of an airway remodeling pathway in the mustard lung model is presented. A schematic workflow of our work is shown in Figure 1.

## Materials and Methods

Static network modeling plays a central role in systems biology. Therefore, the reconstruction process is highly related to our knowledge base in characterizing mustard lung. However, owing to the large amount of data, we need appropriate computational tools for gathering, constructing, and analyzing generated networks and pathways. A schematic workflow of our work is shown in Figure 1 and is described in detail in the main text.

### Data collection

Three chronic sulfur mustard exposed and three unexposed individuals as the control group were selected for gene expression profiling. Airway biopsy was used for microarray gene expression experiment. Differential expression results were achieved during our former work by one way ANOVA test that is equivalent to t-test for two groups. (a P. value <0.01 was accepted as statistically significant after using Bonferroni correction for multiple tests) [19]. Statistical significance was validated using a two-tailed t-test assuming unequal variance, whereby significance was achieved for p<0.01. Therefore, 122 differentially expressed genes were identified by microarray experiment. The raw and process data were deposited in the http://lbb.ut.ac.ir/Download/LBBsoft/Mustard-Lung-Miacroarray. Furthermore, related published gene expression results (real-time PCR) were analyzed for completing the microarray gene list. This investigation attempted for the first time to identify gene profiles using whole human genome microarray chips, so as to recognize potential new target molecules and pathways involved in the pathophysiology of SM-induced airway remodeling. For comparison, there have been several microarray studies on animal models involving analysis of rodent pulmonary tissue [20] and mouse skin [21] after exposure to SM.

Extensive literature surveys were then used to identify and confirm factors (gene, protein and metabolite) involved in airway remodeling. Literature mining was performed using keywords including sulphur mustard, mustard gas, mustard lung, and airway remodeling in Pubmed and google scholar databases without time limitation. Chronic mustard lung studies were selected for further detailed review (File S5). Finally, after removing redundant reports, 50 genes were extracted and merged to the 122 genes identified with microarray experiment (172 genes). iHOP [22] is a Web-based tool and Agilent literature search is a plugin in Cytoscape [23] that were used to automate analysis of abstracts in search for gene names. Finally, data were manually curated. In all cases, the HGNC official gene symbol was used to identify genes and proteins. Gene set annotation enrichment analysis also were performed using DAVID web tools (http://david.abcc.ncifcrf.gov) that provides a set of functional annotation tools for the genes categorized into Gene Ontology (GO) terms.

### Ensemble network and pathway reconstruction

The network and pathways were reconstructed based on the master gene list and the molecular interactions documented in related papers and on-line interaction databases. The Pathway Resource List (http://pathguide.org) is a meta-database that provides an overview of more than 300 web-accessible biological pathway and network databases [24]. Human-specific Interactions of protein–protein interaction (PPI) data were abstracted from Biomolecular Interaction Network Database (BIND) [25], Database of Interacting Proteins (DIP) [26], Mammalian Protein-Protein Interactions Database (MIPS) [27], Human Protein reference Database (HPRD) [28], and Biological General Repository for Interaction Datasets (BioGRID) [29]. Furthermore, Gene regulatory network (GRN) data were obtained from analysis of related microarray experiments and search in GRN database such as GeneMANIA [30] and Search Tool for the Retrieval of Interacting Genes/Proteins (STRING). To achieve the GRN and PPI networks, we entered the mustard lung gene list to these databases and got several GRN and PPI networks for our master gene list. Some of these databases don't accept a gene list; in these cases, o each gene was entered into the database manually, and resulting interactions added to the networks using Cytoscape software. Also, Cytoscape plugins were used for extracting, merging, visualizing, and analyzing unified interactive data. Molecular species in generated networks are represented as nodes and the interactions between these nodes as edges.

Some of the achieved interactions were checked by pathway databases including Protein ANalysis THrough Evolutionary Relationships (PANTHER), Reactome, the National Cancer Institute Pathway Interaction Database (NCI-PID;), the Kyoto Encyclopedia of Genes and Genomes (KEGG), and Pathway Common, as the most widely used databases (table 1).

**Table 1:** Pathway and interactome databases used to identify interaction (edge) between genes in the master list.

Database	Version/date	Graph type	Address
STRING	v8.2	Directed/Undirected	http://string-db.org
Reactome	v43	Directed	www.reactome.org
KEGG	2012	Directed	www.genome.jp/kegg
GeneMania	3.1.1	Undirected	www.genemania.org
NCI-PID	2012	Directed	http://pid.nci.nih.gov
Biocarta	2012	Directed/Undirected	http://www.biocarta.com/
GeneGO	v2.5	Directed	www.genego.com
Pathway Commons	v 3.1.17288	Directed	http://www.pathwaycommons.org
PANTHER	2012	Directed	www.pantherdb.org

Also, generated networks were compiled in Simple Interaction Format (SIF) amenable to Cytoscape for further topological analysis. After merging networks, the topological and statistical significance of the network was calculated using the Network Analyzer plugin in Cytoscape. The degree (connectivity) of nodes, the number of hubs (highly connected nodes), the shortest path lengths between any two nodes, the mean path length (the average of the shortest path lengths) and the network diameter (the maximum of the shortest path lengths) in comparison with random networks (Erdos-Renyi and Barabasi-Albert models; generated by random networks, a plugin in Cytoscape) were analyzed.

Manual Pathway curation provides the most reliable means of extracting information from the literature, databases, and experiments. Thus, based on generated networks (PPI network and GRN), functional modules, signaling pathway databases, and previous knowledge, the airway remodeling pathway map was manually constructed by CellDesigner version 4.2 (www.celldesigner.org) in Systems Biology Markup Language (SBML) format, which provide graphical environment, pathway visualization, and navigation. It can be used for building diagrammatic-based biochemical networks represented as a process flow, with the possibility of running functional simulations.

Based on functional modules derived from mustard lung network, we used signaling pathway databases (Table 1) to map the functional modules to signaling pathways. Each path was identified in different signaling pathways and was manually curated and reconstructed (drawn) using CellDesigner. This stage requires multidisciplinary skills and a priori knowledge.

## Results

### Candidate gene list

Following the workflow described in the Methods section for data retrieval, 172 genes (122 genes from microarray and 50 genes from literature) were selected according to databases, literature mining and related microarray data. The genes that are known to be involved in airway remodeling in sulfur mustard exposed patients are given in the File S1. These genes are annotated and described based on molecular process and functionality in the gene ontology (GO) and other annotation databases.

### Mustard lung networks

We compiled a gene list (nodes) involved in airway remodeling based on PPI resources and literature mining. Briefly, PPI networks are commonly represented in an undirected graph format, with nodes corresponding to proteins and edges corresponding to physical protein-protein interactions. The PPI network obtained from the master list are given in Cytoscape .sif format in File S2. Based on current knowledge of physical interactions in PPI databases, we could identify interactions for just 96 of 172 nodes in the master list. Hence, a PPI network of 96 nodes and 211 edges was generated.

Gene expression data and co-expressed genes have become a useful resource in representing of the molecular state of cells. GRNs are commonly shown in a directed graph. GRNs consist of nodes, representing the expression profile of a particular gene, and edges representing significant associations between expression profiles (for example Pearson correlation). By using our gene list and co-expressed genes (extracted from GRN databases and related microarray data), a mustard lung GRN was generated. This network with 171 nodes and 1002 edges is shown in Cytoscape .sif format in File S3. Also the pdf files of networks are presented in File S6 (PPI) and File S7 (GRN).

**Figure 2:**
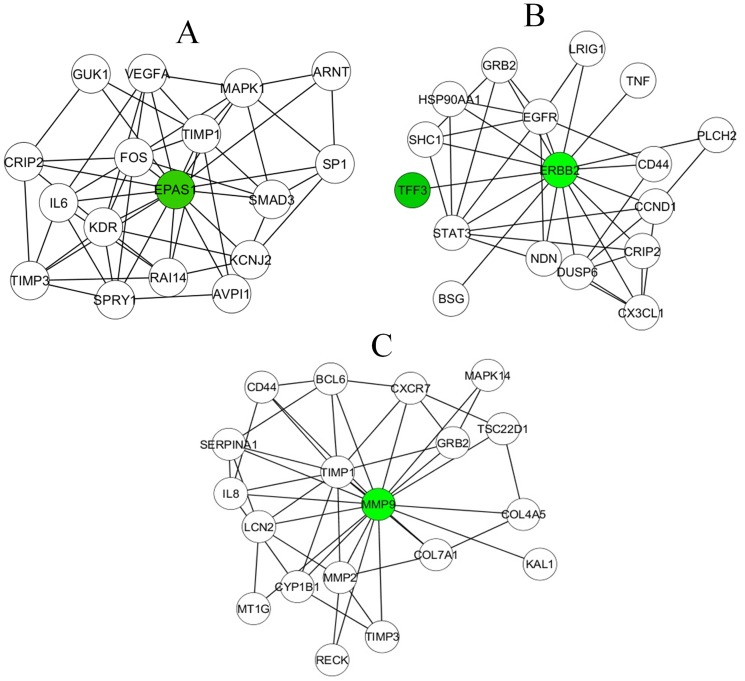
Major disease-risk modules in mustard lung network. Colored nodes are selected based on fold change ranking in microarray gene list and previous reports. They have a central role in these modules as driver nodes. The A, B, and C sub-networks extract from the mustard lung network using Hubba, a plugin in Cytoscape software.

The mustard lung network was created by merging networks. The complex network is likely composed of several sub-networks or functional modules contributing to various diverse biological processes in mustard lung disease. Three major functional modules are illustrated in figure 2.

### Proposed signaling pathway

**Figure 3:**
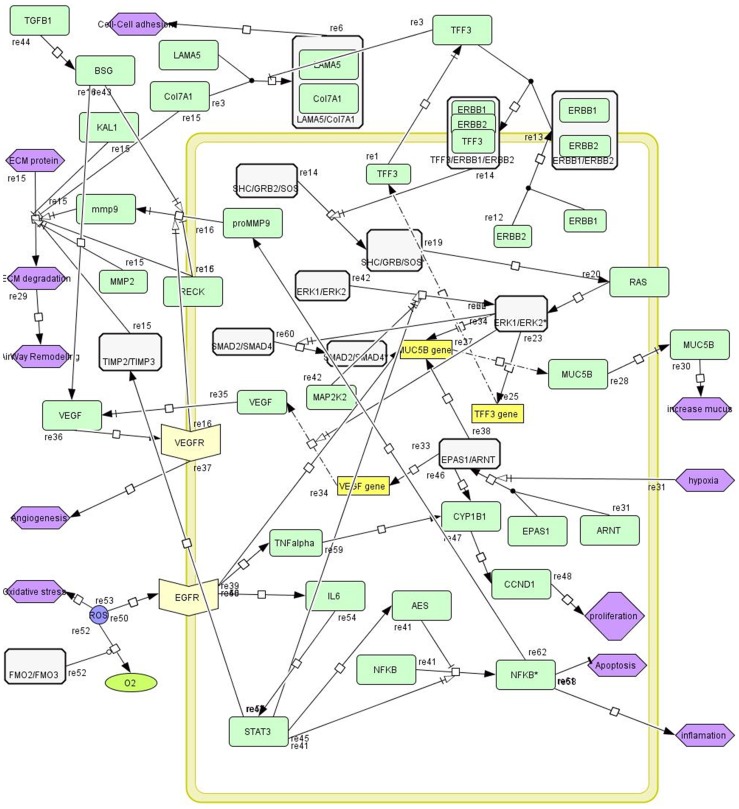
The proposed pathway of airway remodeling in mustard lung. Pathway nodes are indicated by color coding; Green: single protein; White: protein complex; Yellow box: gene; Bisque: receptor protein; Purple: biological process. The pathway illustrates several paths such as ERK/MAPK, ERBB1/ERBB2 complex activation via TFF3, and EPAS1/ARNT transcription factor activation. Different paths were extracted from pathway databases (table 1) and then were curated and reconstructed manually in the CellDesigner graphical interface.

Pathway extraction or static modeling of networks (PPI network and GRN) has become a very active area of research. Figure 3 shows a schematic pathway that is generated by CellDesigner in SBML format (File S4). This pathway has been extracted from the generated mustard lung network. There are several signaling paths such as ERK/MAPK, EGFR activation, and EPAS1/ARNT paths in this disease pathway that have high crosstalk together.

### Network topology analysis

**Figure 4:**
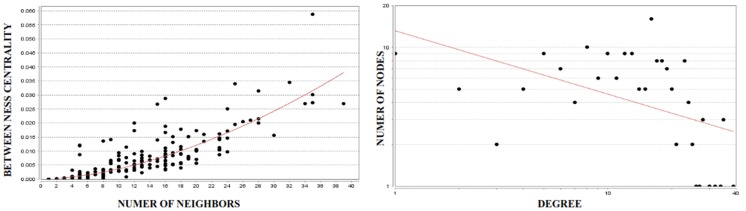
Analysis of mustard lung network with 172 nodes and 1169 edges. These graphs were generated using NetworkAnalyzer plugin of Cytoscape. The scatter plot of betweenness centrality vs. number of neighbors (left) indicates that a limited number of nodes control the information flow between other nodes within the biological network. This means that a limited number of nodes with high interactions (hubs) control other nodes with lower interactions. The node degree distribution (right) shows that the network is scale-free considering the power-law degree distribution *P(k)∼k^γ^* (fitting result is γ = 0. 790 and R-squared = 0.667). This means that the mustard lung network is a biological network which differs from random networks.

Network parameters were performed by using NetworkAnalyzer plugin of Cytoscape for the mustard lung network. Topological analysis of a network identifies the global qualitative properties of the system. Network topology is used to provide the significance of a node in communicating with other nodes. Scale-free networks share two important functional characteristics. First, they are differently sensitive to damage. So if a small, peripheral node stops functioning, the network is very likely to continue working without problem. By contrast, if a hub is damaged, the functionality of the entire network is likely to be jeopardized. These topological characteristics are seen in biological networks [31]. In other words, if a hub node such as MMP9 is closed, most of the nodes and edges will be affected. Since biological networks satisfy power-law degree distribution, we have checked the mustard lung network for basic network properties. Degree distribution of a scale-free network having *k* connections to other nodes satisfies the following relation [32]:
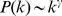
where *γ* is power-law parameter. For mustard lung network, we have applied curve fitting to aforementioned relation and calculated values of *γ* and *r^2^* (coefficient of determination or R-squared). According to the *r^2^*, the results show that mustard lung network is scale-free (Figure 4-right). The betweenness centrality *Cb(n)* of a node n is computed as follows:
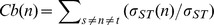
where *s* and *t* are nodes in the network different from *n*, *σ_st_* denotes the number of shortest paths from *s* to *t*, and *σ_st_* (*n*) is the number of shortest paths from *s* to *t* that *n* lies on (Figure 4-left).

Topological parameters such as the average clustering coefficient, topological coefficient, average degree and power law distribution of degrees and betweenness centrality have been assessed to predict the topology of the networks. The distribution of clustering coefficient is an important characteristic of biological scale-free networks. The clustering coefficient ***C_n_*** of a node *n* is defined as *C_n_* = 2*e_n_*/(*k_n_*(*k_n_*-1)), where *k_n_* is the number of neighbors of *n* and *e_n_* is the number of connected pairs between all neighbors of *n*. Therefore, the clustering coefficient, characteristic path length, network centralization and network density of the Mustard lung network **were** compared with simulated randomized model networks (Erdos-Renyi and Barabasi-Albert models; generated by random networks, a plugin in Cytoscape) as shown in Table 2. The general structure of the network is far from random as shown by the comparison with the simulated network. In particular, the clustering coefficient of the Mustard lung network greatly differs from random network. These data indicate that the interactome possesses its own peculiar structure, and not a random one, which is a sign of a highly organized architecture.

**Table 2:** Basic network parameters of the two generated networks, compared with simulated randomized model networks.

	Mustard lung network	Simulated Barabasi Albert model, (scale free)	Simulated Erdos-Renyi model
Number of nodes	172	172	172
Clustering coefficient	0.208	0.127	0.054
Characteristic path length	2.419	3.206	2.610
Network density	0.079	0.023	0.051
Network centralization	0.150	0.225	0.049

The clustering coefficient (table 2) is the average of the clustering coefficient for all nodes in each network. The characteristic path length gives the expected distance between two connected nodes in each network and the network density and network centralization show how densely the network is populated with edges and reflect the tendency of a network to contain hub nodes.

## Discussion

Cellular signaling is a complex system governing the events in the organism that can be considered as communication both within and between cells. Airway remodeling occurs in many chronic lung disorders. The bronchial epithelium, sub-epithelial myofibroblasts and ASM (airway smooth muscle) cells are the major cell types involved in tissue repair processes in the airway tissue. Highly dynamic interactions and crosstalk between these resident cells via signaling proteins through autocrine or paracrine mechanisms dictate how tissue repair should progress. Signaling proteins from different pathways may interact directly (e.g. by phosphorylation) or influence each other indirectly (e.g. via regulation of gene expression). One component may also act in more than one pathway. Such cross-talk events can result in unexpected behavior.

For the first time, we reconstructed an enriched network and a pathway that comprehensively catalogs major signaling pathways in the field of airway remodeling associated with sulfur mustard exposure. Disease pathway construction is essential to decipher complex regulatory patterns in deranged cellular processes. Therefore, identifying disease networks and pathways is important for understanding the pathology of disease and improving clinical diagnosis and treatment. Based on network topology analysis in comparison with random network, the generated networks have a larger clustering coefficient, higher network density, higher network centralization, and shorter characteristic path length than generated random networks. All of these features reveal a high degree of connectivity found in biological scale free networks. This type of network with high levels of global connectivity and high-degree hubs is often found in biological systems that respond to changing external conditions [33]. However, while this system design is useful for normal tissue, in abnormal conditions such as mustard lung, the effects of network disruptions can spread rapidly. We have assembled a comprehensive map of putative regulators of airway remodeling in human airway epithelial layer of mustard lung. This pathway is composed of complexes of physically interacting genes and proteins, which are investigated with molecular biology approaches in genomics, proteomics, and metabolomics.

Previous studies have shown various signaling processes such as: angiogenesis, inflammation, oxidative stress, mucous secretion, apoptosis/antiapoptosis, and ECM degradation in airway remodeling [15], [34], [35]. Clinical and pathological signs of mustard lung also confirmed these processes. Disruption of a pathway may be a good thing. Thus, the detailed understanding of molecular pathways is an important step in diagnosis and treatment of disease. Eventually, it can help to determine biomarkers for complex diseases, which may lead to new approach for treatment and diagnosis [36], [37].

Several signaling paths were identified in the generated mustard lung pathway, including EPAS1/ARNT path, ERK/MAPK path, MMPs path, and EGFR path. These highly connected paths can lead to various processes and phenotypes. Details and functions of these processes are discussed below.

### Extracellular Matrix and adhesion molecules regulation

The extracellular matrix (ECM) is a complex composition of different combinations of collagens, proteoglycans, hyaluronic acid, laminin, fibronectin, and many other glycoproteins, including proteolytic enzymes involved in the degradation and remodeling of the extracellular matrix. One of the major roles of ECM is to serve as a structural framework for cell attachment and migration. Among proteases, matrix metalloproteinases (MMPs) are extracellular degrading enzymes that play a critical role in degrading components of the ECM and epithelial cell junctions, cancer metastasis, and liberation of tethered growth and chemotactic factors in response to wounding [38]. One of the lung disorders that has both inflammatory and remodeling features is mustard lung. Imbalance between levels of MMPs and TIMPs in a particular location can lead to accumulation of ECM protein at that site. The most well-known profibrotic cytokine TGF-β is known to crosstalk with the ECM pathway. Also, TGF-β signaling has a critical role in activation of angiogenesis [39].

### Mucus hyper-secretion

Mucus hyper-secretion is a major pathophysiologic feature in chronic inflammatory airway diseases. Trefoil Factor3 (TFF3) is one starting point in the airway remodeling pathway as shown in figure 3. Trefoil family has three homologous (TFF1, TFF2, and TFF3) [40], [41]. Although the exact function of TFF3 in the respiratory tract remains largely unknown, experiments using recombinant human TFFs (hTFF) show that they may serve several roles in maintaining intestinal homeostasis such as by potentiating intestinal permeability, increasing the mucous integrity through mucin interactions, or directing epithelial restitution by promoting cellular migration. Also, Greeley et al. have shown that TFF1, TFF2, and TFF3 have roles in proliferation, as well as migration, cell death and differentiation phases of airway epithelial repair [42]. In our pathway, TFF3 acts as a ligand and binds to ErbB1/ErbB2 complex (ERBB1 also known as EGFR). Heterodimerization of ErbB1/ErbB2 stimulate ERK kinase cascade. ErbB1 and ErbB2 recruit Son of Sevenless homolog (SOS) via adaptor protein GRB2 and SHC transforms protein (Shc), respectively. SOS is a guanine-nucleotide exchange factor for small GTPases, including H-Ras. Following this activation, the MAPK/ERK path is activated and we have shown MAP2K2 over-expression that has a critical role in ERK pathway. Then, this cascade continues with the activation of downstream pathways. At the end of the pathway, Muc5B gene is expressed which leads to enhanced mucus secretion. Also, this cascade causes expression of TFF3 gene, leading to increased TFF3 protein in airway tissue.

### Angiogenesis

Angiogenesis is a hallmark in the pathology of many diseases, including cancer, ischemia, atherosclerosis, and inflammatory diseases. Angiogenesis has additional roles in normal development and physiological processes in adults, including wound healing and tissue regeneration. Most previous studies have shown that hypoxia inducible factor 1alpha (HIF1A) and endothelial PAS domain protein 1 (EPAS1) transcription factors promote the transcription of vascular endothelial growth factor (VEGF) when they interact with aryl hydrocarbon receptor nuclear translocator (ARNT). Moreover, *in-vitro* experiments showed that EPAS1 (or HIF2A) mRNA is expressed in the epithelial cells of pulmonary alveoli [43]. This complex binds to the promoters and then activates target genes involved in proliferation and differentiation, such as MMP9, VEGFA, CYP1B1, CCND1, and MUC5B. The two subunits EPAS1/ARNT are together forming a transcription factor that regulates expression of VEGF that enhances angiogenesis in airway tissue. Also EPAS1/ARNT regulates more than 100 genes which regulate important mechanisms such as angiogenesis, apoptosis, and anaerobic metabolism. In mucus hyper-secretion and hypoxia, the EPAS1/ARNT complex is activated, and thus expression of downstream genes is increased as a result. EPAS1 protein increases its own promoter activity as well as the transcription of the VEGF gene.

It has also been noted that in prolonged hypoxia in lung epithelial cells, the up-regulation of HIF1α was transient, whereas increases in HIF2α were sustained [44]. A transient up-regulation of HIF1α could not be ruled out in these conditions, but the expression of HIF1α was significantly lower than HIF2α under all conditions.

CXCL17 is a chemokine involved in tumor angiogenesis and is an anti-inflammatory factor that we have also observed to be over-expressed in mustard lung. CXCL17 induces the production of proangiogenic factors such as VEGFA. Also, CXCL17 is expressed in some aggressive types of gastrointestinal, lung and breast cancer cells [45].

TGF-beta1 is involved in extracellular matrix regulation, leading to airway remodeling in chronic airway diseases. Willems-Widyastuti et al. proved that TGF-beta1 acting through ECM regulation could also play a critical role in bronchial angiogenesis and vascular remodeling via VEGF pathway in asthma [46].

### Inflammation and oxidative stress

Oxidative stress plays a pivotal role in the pathogenesis of many chronic inflammatory lung diseases, especially in mustard lung. Oxidative stress induced cellular and molecular changes in the airway epithelium layer may significantly contribute to the pathogenesis of chronic inflammatory airway disorders and induce apoptotic or inflammatory responses in the lower respiratory tract [47].

Airway cells are constantly challenged by environmental (e.g. xenobiotics, UV, and drugs) and endogenous stressors (e.g. reactive oxygen species (ROS), hydroproxides, carbonyls, and quinones). If unchecked, these stresses lead to airway and lung inflammation. FMO2/FMO3 complex is one of the key enzymes that catalyze ROS to O2. With decreasing expression of this complex, ROS increases and therefore, leads to oxidative stress and inflammation. In addition, ROS is a second messenger required for regulating MUC5B expression and mucus secretion via EGFR signaling path [48].

### Apoptosis and anti-apoptosis regulation

Apoptosis (programmed cell death) and necrosis are the two major types of cell death. The process of apoptosis is triggered by a diverse range of cellular signals. Either the extrinsic or intrinsic death pathways can lead to apoptosis. Extracellular signals include toxins, hormones, growth factors, cytokines, nitric oxide, heat, radiation, nutrient deprivation, viral infection, hypoxia, the binding of nuclear receptors by glucocorticoids, or increased intracellular calcium concentration can damage DNA or cause cellular stress, triggering the release of intracellular apoptotic signals. There are some genes in File S3 that are connected to apoptosis, including CRIP2, PACS-2, and IFI35. Over-expression of CRIP2 induces apoptosis through activation of caspases 3 and 9 [49]. CRIP2 acts as a transcription repressor of the nuclear factor-κB-mediated proangiogenic cytokine expression [50]. TRAIL/DR5 triggers PACS-2 to traffic Bim and Bax to lysosomes to release cathepsin B and induce apoptosis [51] and IFI35 is induced by IFN-gamma [52]. Some of the identified factors (such as TSPAN8, CXCL17, CRIP2, STARD10, CSNK2A1) and pathways (i.e. apoptosis, TGFβ, VEGF and ERK signaling) are known to participate in cancer as well as their identification here linked with airway remodeling in mustard lung. Our data suggest that cancer and airway remodeling share several overlapping processes.

This work has a few limitations. First, airway biopsy sampling is an invasive technique (especially for normal group), and most of the chronic mustard patients have about 25-year difficulties in their life style and strongly refuse to be included in the study. Combined with various exclusion criteria including age, smoking, and lung performance, the study population was quite small, thus gene profiling of each biopsy sample was performed in duplicate for further accuracy. Second, our analysis necessarily includes data from mouse models of airway remodeling which may or may not be representative of human disease.

## Conclusions

Our findings demonstrate that the analysis and modeling of complex biological networks are beyond the capabilities of existing computational techniques that are needed for this type of scientific endeavor. Therefore, manual curation and pathway reconstruction was followed after computational results. Current diagnosis of the airway remodeling diseases, in particular mustard lung and COPD, is mainly based on a combination of lung function evaluation and an observation of symptoms. There is no sole and exact clinical or laboratory test available. This highlights the need for biomarkers to diagnose disease or identify disease phenotypes. Therefore, the identification of key candidate genes, and their roles in regulating pathways, shows the need for bringing systems biology to the clinic as a powerful new approach. TFF3, ERBB2, EPAS1, and COL7A1 are the candidate factors identified here as potentially centrally involved in the pathophysiology of airway remodeling in mustard lung. The regulation of these proteins may be potentially useful in the treatment of airway remodeling. Finally, a comprehensive understanding of biological pathways can aid in the development of drugs to target specific cellular mechanisms while avoiding unwanted side effects.

## Supporting Information

File S1 **List of Genes that are known to be involved in airway remodeling in sulfur mustard exposed patients and their propertises.**
(XLS)

File S2 **The PPI network obtained from the master gene list are given in Cytoscape .sif format.**
(SIF)

File S3 **The GRN network with 171 nodes and 1002 edges is shown in Cytoscape .sif format.**
(SIF)

File S4 **The proposed mustard lung pathway that is generated by CellDesigner in SBML format.**
(XML)

File S5 **The reference list is used in literature mining for gene selection.**
(DOCX)

File S6 **The pdf file of PPI network.**
(PDF)

File S7 **The pdf file of GRN network.**
(PDF)

## References

[1] Tschumperlin DJ, Drazen JM (2001) Mechanical stimuli to airway remodeling. American journal of respiratory and critical care medicine 164: S90–S94. 11734475 10.1164/ajrccm.164.supplement_2.2106060

[2] Thomason JW, Rice TW, Milstone AP (2003) Bronchiolitis obliterans in a survivor of a chemical weapons attack. JAMA 290: 598–599. 12902361 10.1001/jama.290.5.598

[3] Ghanei M, Mokhtari M, Mohammad MM, Aslani J (2004) Bronchiolitis obliterans following exposure to sulfur mustard: chest high resolution computed tomography. Eur J Radiol 52: 164–169. 15489074 10.1016/j.ejrad.2004.03.018

[4] Ghanei M, Harandi AA (2007) Long term consequences from exposure to sulfur mustard: a review. Inhal Toxicol 19: 451–456. 17365048 10.1080/08958370601174990

[5] Chen L, Li W, Zhang L, Wang H, He W, et al (2011) Disease gene interaction pathways: a potential framework for how disease genes associate by disease-risk modules. PLoS One 6: e24495. 21915342 10.1371/journal.pone.0024495 PMC3167857

[6] Bugrim A, Nikolskaya T, Nikolsky Y (2004) Early prediction of drug metabolism and toxicity: systems biology approach and modeling. Drug Discov Today 9: 127–135. 14960390 10.1016/S1359-6446(03)02971-4

[7] Kitano H, Funahashi A, Matsuoka Y, Oda K (2005) Using process diagrams for the graphical representation of biological networks. Nat Biotechnol 23: 961–966. 16082367 10.1038/nbt1111

[8] Kann MG (2007) Protein interactions and disease: computational approaches to uncover the etiology of diseases. Brief Bioinform 8: 333–346. 17638813 10.1093/bib/bbm031

[9] Kitano H (2002) Computational systems biology. Nature 420: 206–210. 12432404 10.1038/nature01254

[10] Wheelock CE, Wheelock AM, Kawashima S, Diez D, Kanehisa M, et al (2009) Systems biology approaches and pathway tools for investigating cardiovascular disease. Mol Biosyst 5: 588–602. 19462016 10.1039/b902356a

[11] Hallock P, Thomas MA (2012) Integrating the Alzheimer's disease proteome and transcriptome: a comprehensive network model of a complex disease. OMICS 16: 37–49. 22321014 10.1089/omi.2011.0054 PMC3275800

[12] Wachi S, Yoneda K, Wu R (2005) Interactome-transcriptome analysis reveals the high centrality of genes differentially expressed in lung cancer tissues. Bioinformatics 21: 4205–4208. 16188928 10.1093/bioinformatics/bti688 PMC4631381

[13] King JY, Ferrara R, Tabibiazar R, Spin JM, Chen MM, et al (2005) Pathway analysis of coronary atherosclerosis. Physiol Genomics 23: 103–118. 15942018 10.1152/physiolgenomics.00101.2005

[14] Lesnick TG, Papapetropoulos S, Mash DC, Ffrench-Mullen J, Shehadeh L, et al (2007) A genomic pathway approach to a complex disease: axon guidance and Parkinson disease. PLoS Genet 3: e98. 17571925 10.1371/journal.pgen.0030098 PMC1904362

[15] Ghanei M, Harandi AA (2011) Molecular and cellular mechanism of lung injuries due to exposure to sulfur mustard: a review. Inhal Toxicol 23: 363–371. 21639706 10.3109/08958378.2011.575413 PMC3128827

[16] Ghanei M, Adibi I, Farhat F, Aslani J (2008) Late respiratory effects of sulfur mustard: how is the early symptoms severity involved? Chron Respir Dis 5: 95–100. 18539723 10.1177/1479972307087191

[17] Ghabili K, Agutter PS, Ghanei M, Ansarin K, Shoja MM (2010) Mustard gas toxicity: the acute and chronic pathological effects. J Appl Toxicol 30: 627–643. 20836142 10.1002/jat.1581

[18] Mishra NC, Rir-sima-ah J, Grotendorst GR, Langley RJ, Singh SP, et al (2012) Inhalation of sulfur mustard causes long-term T cell-dependent inflammation: possible role of Th17 cells in chronic lung pathology. Int Immunopharmacol 13: 101–108. 22465472 10.1016/j.intimp.2012.03.010 PMC3340497

[19] Najafi A, Masoudi-Nejad A, Imani Fooladi AA, Ghanei M, Nourani MR (2014) Microarray gene expression analysis of the human airway in patients exposed to sulfur mustard. J Recept Signal Transduct Res 1–7. 10.3109/10799893.2014.896379 24823320

[20] Dillman JF 3rd, Phillips CS, Dorsch LM, Croxton MD, Hege AI, et al (2005) Genomic analysis of rodent pulmonary tissue following bis-(2-chloroethyl) sulfide exposure. Chem Res Toxicol 18: 28–34. 15651846 10.1021/tx049745z

[21] Rogers JV, Choi YW, Kiser RC, Babin MC, Casillas RP, et al (2004) Microarray analysis of gene expression in murine skin exposed to sulfur mustard. J Biochem Mol Toxicol 18: 289–299. 15674843 10.1002/jbt.20043

[22] Fernandez JM, Hoffmann R, Valencia A (2007) iHOP web services. Nucleic Acids Res 35: W21–26. 17485473 10.1093/nar/gkm298 PMC1933131

[23] Smoot ME, Ono K, Ruscheinski J, Wang PL, Ideker T (2011) Cytoscape 2.8: new features for data integration and network visualization. Bioinformatics 27: 431–432. 21149340 10.1093/bioinformatics/btq675 PMC3031041

[24] Bader GD, Cary MP, Sander C (2006) Pathguide: a pathway resource list. Nucleic Acids Res 34: D504–506. 16381921 10.1093/nar/gkj126 PMC1347488

[25] Bader GD, Betel D, Hogue CW (2003) BIND: the Biomolecular Interaction Network Database. Nucleic Acids Res 31: 248–250. 12519993 10.1093/nar/gkg056 PMC165503

[26] Xenarios I, Salwinski L, Duan XJ, Higney P, Kim SM, et al (2002) DIP, the Database of Interacting Proteins: a research tool for studying cellular networks of protein interactions. Nucleic Acids Res 30: 303–305. 11752321 10.1093/nar/30.1.303 PMC99070

[27] Pagel P, Kovac S, Oesterheld M, Brauner B, Dunger-Kaltenbach I, et al (2005) The MIPS mammalian protein-protein interaction database. Bioinformatics 21: 832–834. 15531608 10.1093/bioinformatics/bti115

[28] Peri S, Navarro JD, Amanchy R, Kristiansen TZ, Jonnalagadda CK, et al (2003) Development of human protein reference database as an initial platform for approaching systems biology in humans. Genome Res 13: 2363–2371. 14525934 10.1101/gr.1680803 PMC403728

[29] Chatr-Aryamontri A, Breitkreutz BJ, Heinicke S, Boucher L, Winter A, et al (2013) The BioGRID interaction database: 2013 update. Nucleic Acids Res 41: D816–823. 23203989 10.1093/nar/gks1158 PMC3531226

[30] Mostafavi S, Ray D, Warde-Farley D, Grouios C, Morris Q (2008) GeneMANIA: a real-time multiple association network integration algorithm for predicting gene function. Genome Biol 9 Suppl 1: S4. 10.1186/gb-2008-9-s1-s4 PMC2447538 18613948

[31] Agusti A, Sobradillo P, Celli B (2011) Addressing the complexity of chronic obstructive pulmonary disease: from phenotypes and biomarkers to scale-free networks, systems biology, and P4 medicine. Am J Respir Crit Care Med 183: 1129–1137. 21169466 10.1164/rccm.201009-1414PP

[32] Junker BH, Schreiber F (2011) Analysis of Biological Networks: Wiley.

[33] Zhu X, Gerstein M, Snyder M (2007) Getting connected: analysis and principles of biological networks. Genes Dev 21: 1010–1024. 17473168 10.1101/gad.1528707

[34] Adelipour M, Imani Fooladi AA, Yazdani S, Vahedi E, Ghanei M, et al (2011) Smad molecules expression pattern in human bronchial airway induced by sulfur mustard. Iran J Allergy Asthma Immunol 10: 147–154. 21891820

[35] Yazdani S, Karimfar MH, Imani Fooladi AA, Mirbagheri L, Ebrahimi M, et al (2011) Nuclear factor kappaB1/RelA mediates the inflammation and/or survival of human airway exposed to sulfur mustard. J Recept Signal Transduct Res 31: 367–373. 21929290 10.3109/10799893.2011.602415

[36] Barabási AL, Gulbahce N, Loscalzo J (2011) Network medicine: a network-based approach to human disease. Nat Rev Genet 12: 56–68. 21164525 10.1038/nrg2918 PMC3140052

[37] Loscalzo J (2011) Systems biology and personalized medicine: a network approach to human disease. Proc Am Thorac Soc 8: 196–198. 21543801 10.1513/pats.201006-041MS

[38] Snyder JC, Zemke AC, Stripp BR (2009) Reparative capacity of airway epithelium impacts deposition and remodeling of extracellular matrix. Am J Respir Cell Mol Biol 40: 633–642. 18978301 10.1165/rcmb.2008-0334OC PMC2689915

[39] P. Carmeliet RKJ (2011) Molecular mechanisms and clinical applications of angiogenesis. Nature 473: 298–307. 21593862 10.1038/nature10144 PMC4049445

[40] Taupin D, Podolsky DK (2003) Trefoil factors: initiators of mucosal healing. Nature Reviews Molecular Cell Biology 4: 721–732. 14506475 10.1038/nrm1203

[41] Royce SG, Lim C, Muljadi RC, Samuel CS, Ververis K, et al (2013) Trefoil factor-2 reverses airway remodeling changes in allergic airways disease. Am J Respir Cell Mol Biol 48: 135–144. 22652198 10.1165/rcmb.2011-0320OC

[42] Greeley MA, Van Winkle LS, Edwards PC, Plopper CG (2010) Airway trefoil factor expression during naphthalene injury and repair. Toxicol Sci 113: 453–467. 19880587 10.1093/toxsci/kfp268 PMC2807036

[43] Sato M, Tanaka T, Maeno T, Sando Y, Suga T, et al (2002) Inducible expression of endothelial PAS domain protein-1 by hypoxia in human lung adenocarcinoma A549 cells. Role of Src family kinases-dependent pathway. Am J Respir Cell Mol Biol 26: 127–134. 11751212 10.1165/ajrcmb.26.1.4319

[44] Uchida T, Rossignol F, Matthay MA, Mounier R, Couette S, et al (2004) Prolonged hypoxia differentially regulates hypoxia-inducible factor (HIF)-1alpha and HIF-2alpha expression in lung epithelial cells: implication of natural antisense HIF-1alpha. J Biol Chem 279: 14871–14878. 14744852 10.1074/jbc.M400461200

[45] Matsui A, Yokoo H, Negishi Y, Endo-Takahashi Y, Chun NA, et al (2012) CXCL17 expression by tumor cells recruits CD11b+Gr1 high F4/80- cells and promotes tumor progression. PLoS One 7: e44080. 22952881 10.1371/journal.pone.0044080 PMC3430639

[46] Willems-Widyastuti A, Alagappan VK, Arulmani U, Vanaudenaerde BM, Boer WId, et al (2011) Transforming growth factor-1 induces angiogenesis in vitro via VEGF production in human airway smooth muscle cells. 22053695

[47] Kim HJ, Ryu JH, Kim CH, Lim JW, Moon UY, et al (2010) Epicatechin gallate suppresses oxidative stress-induced MUC5AC overexpression by interaction with epidermal growth factor receptor. Am J Respir Cell Mol Biol 43: 349–357. 19855084 10.1165/rcmb.2009-0205OC

[48] Fischer BM, Voynow JA (2002) Neutrophil elastase induces MUC5AC gene expression in airway epithelium via a pathway involving reactive oxygen species. Am J Respir Cell Mol Biol 26: 447–452. 11919081 10.1165/ajrcmb.26.4.4473

[49] Lo PHY, Ko JMY, Yu ZY, Law S, Wang LD, et al (2012) The LIM domain protein, CRIP2, promotes apoptosis in esophageal squamous cell carcinoma. Cancer Lett 316: 39–45. 22154084 10.1016/j.canlet.2011.10.020

[50] Cheung AK, Ko JM, Lung HL, Chan KW, Stanbridge EJ, et al (2011) Cysteine-rich intestinal protein 2 (CRIP2) acts as a repressor of NF-kappaB-mediated proangiogenic cytokine transcription to suppress tumorigenesis and angiogenesis. Proc Natl Acad Sci U S A 108: 8390–8395. 21540330 10.1073/pnas.1101747108 PMC3100921

[51] Werneburg NW, Bronk SF, Guicciardi ME, Thomas L, Dikeakos JD, et al (2012) Tumor necrosis factor-related apoptosis-inducing ligand (TRAIL) protein-induced lysosomal translocation of proapoptotic effectors is mediated by phosphofurin acidic cluster sorting protein-2 (PACS-2). Journal of Biological Chemistry 287: 24427–24437. 22645134 10.1074/jbc.M112.342238 PMC3397868

[52] Rebane A, Zimmermann M, Aab A, Baurecht H, Koreck A, et al (2012) Mechanisms of IFN-gamma-induced apoptosis of human skin keratinocytes in patients with atopic dermatitis. J Allergy Clin Immunol 129: 1297–1306. 22445417 10.1016/j.jaci.2012.02.020

