# Supplementary material for: Pathway Reconstruction of Airway Remodeling in Chronic Lung Diseases: A Systems Biology Approach
Source: PLoS One. 2014 Jun 30;9(6):e100094. doi: 10.1371/journal.pone.0100094 (PMC4076832; doi:10.1371/journal.pone.0100094)
Supplement: File S5 — The reference list is used in literature mining for gene selection. (DOCX) [file pone.0100094.s005.docx]

**Literature mining**

| **Reference** | **genes** |
| --- | --- |
| Adelipour, M., et al. (2011). "Smad molecules expression pattern in human bronchial airway induced by sulfur mustard." Iran J Allergy Asthma Immunol **10**(3): 147-154. | TGF-beta, Smad2, Smad3, Smad4 and Smad7 |
| Aghanouri, R., et al. (2004). "Fibrogenic cytokine levels in bronchoalveolar lavage aspirates 15 years after exposure to sulfur mustard." Am J Physiol Lung Cell Mol Physiol **287**(6): L1160-1164. | TGF-beta, IFN-gamma |
| Ebrahimi, M., et al. (2010). "Discrepancy between mRNA and Protein Expression of Neutrophil Gelatinase-Associated Lipocalin in Bronchial Epithelium Induced by Sulfur Mustard." J Biomed Biotechnol **2010**: 823131. | ROS, NGAL, Lcn2 |
| Ghanei, M., et al. (2007). "Correlation of sulfur mustard exposure and tobacco use with expression (immunoreactivity) of p53 protein in bronchial epithelium of Iranian "mustard lung" patients." Mil Med **172**(1): 70-74. | p53 |
| Jafari, M. and M. Ghanei (2010). "Evaluation of plasma, erythrocytes, and bronchoalveolar lavage fluid antioxidant defense system in sulfur mustard-injured patients." Clin Toxicol (Phila) **48**(3): 184-192. | Superoxide dismutase, catalase, and glutathione peroxidase |
| Karami, A., et al. (2011). "Vascular endothelial growth factor in bronchoalveolar lavage fluid in sulfur mustard exposed lung patients." Oman Med J **26**(2): 118-121. | VEGF |
| Khaheshi, I., et al. (2011). "Loss of expression of TGF-betas and their receptors in chronic skin lesions induced by sulfur mustard as compared with chronic contact dermatitis patients." BMC Dermatol **11**: 2. | TGF-beta |
| Mishra, N. C., et al. (2012). "Inhalation of sulfur mustard causes long-term T cell-dependent inflammation: possible role of Th17 cells in chronic lung pathology." Int Immunopharmacol **13**(1): 101-108. | TGF-beta, IL-1beta, TNF-alpha, IL-2, IL-6, CCL2, CCL3, CCL11, and CXCL1 |
| Nourani, M. R., et al. (2011). "Sulfur mustard induces expression of metallothionein-1A in human airway epithelial cells." Int J Gen Med **4**: 413-419. | Metallothioneins |
| Nourani, M. R., et al. (2009). "HO1 mRNA and Protein do not Change in Parallel in Bronchial Biopsies of Patients After Long Term Exposure to Sulfur Mustard." Gene Regul Syst Bio **4**: 83-90. | ROS and Heme oxygenases |
| Pourfarzam, S., et al. (2009). "Serum levels of IL-8 and IL-6 in the long term pulmonary complications induced by sulfur mustard: Sardasht-Iran Cohort Study." Int Immunopharmacol **9**(13-14): 1482-1488. | IL-6, IL-8 |
| Shohrati, M., et al. (2008). "Activity and function in lung injuries due to sulphur mustard." Biomarkers **13**(7): 728-733. | superoxide dismutase (SOD) |
| Yazdani, S., et al. (2011). "Nuclear factor kappaB1/RelA mediates the inflammation and/or survival of human airway exposed to sulfur mustard." J Recept Signal Transduct Res **31**(5): 367-373. | Nuclear factor kappaB1/RelA |
| Churg, A., et al. (2006). "Cigarette smoke drives small airway remodeling by induction of growth factors in the airway wall." Am J Respir Crit Care Med **174**(12): 1327-1334. | CTGF, (TGF)-beta(1), (PDGF) |
| Comhair, S. A., et al. (2005). "Superoxide dismutase inactivation in pathophysiology of asthmatic airway remodeling and reactivity." Am J Pathol **166**(3): 663-674. | superoxide dismutase (SOD) |
| Gao, G. X., et al. (2009). "Effect of Astragali-Cordyceps Mixtura on TGF-beta/Smad signal pathway in the lung of asthma airway remodeling." J Ethnopharmacol **125**(1): 68-74. | TGF-beta1, Smad3 and Smad7 |
| Lappalainen, U., et al. (2005). "Interleukin-1beta causes pulmonary inflammation, emphysema, and airway remodeling in the adult murine lung." Am J Respir Cell Mol Biol **32**(4): 311-318. | CXCL1, MIP-2 (CXCL2), MMP-9, MMP-12, and IL-1beta |
| Leigh, R., et al. (2008). "Human rhinovirus infection enhances airway epithelial cell production of growth factors involved in airway remodeling." J Allergy Clin Immunol **121**(5): 1238-1245 e1234. | VEGF |
| Makinde, T., et al. (2007). "The regulatory role of TGF-beta in airway remodeling in asthma." Immunol Cell Biol **85**(5): 348-356. | TGF-beta |
| Ohbayashi, H. and K. Shimokata (2005). "Matrix metalloproteinase-9 and airway remodeling in asthma." Curr Drug Targets Inflamm Allergy **4**(2): 177-181. | MMP-9, TGF beta |
| Royce, S. G., et al. (2013). "Trefoil factor-2 reverses airway remodeling changes in allergic airways disease." Am J Respir Cell Mol Biol **48**(1): 135-144. | TFFs |
| Royce, S. G., et al. (2013). "Trefoil factor-2 reverses airway remodeling changes in allergic airways disease." Am J Respir Cell Mol Biol **48**(1): 135-144. | TFFs |
| Sun, Y., et al. (2012). "[Effects of budesonide on HIF-1alpha and VEGF expression and airway remodeling in an asthmatic mouse model]." Zhongguo Dang Dai Er Ke Za Zhi **14**(8): 622-627. | HIF-1alpha, VEGF |
| Zhang, K., et al. (2008). "[Effects of Shenqi Bufei Tang on expessions of NF-kappaB, MMP-9 and TIMP-1 in airway remodeling of COPD rat model with lung-Qi deficiency syndrome]." Zhongguo Zhong Yao Za Zhi **33**(18): 2129-2132. | NF-kappaB, MMP-9 and TIMP-1 |
