# Supplementary figures and images for: Pathway Reconstruction of Airway Remodeling in Chronic Lung Diseases: A Systems Biology Approach
Source: PLoS One. 2014 Jun 30;9(6):e100094. doi: 10.1371/journal.pone.0100094 (PMC4076832; doi:10.1371/journal.pone.0100094)

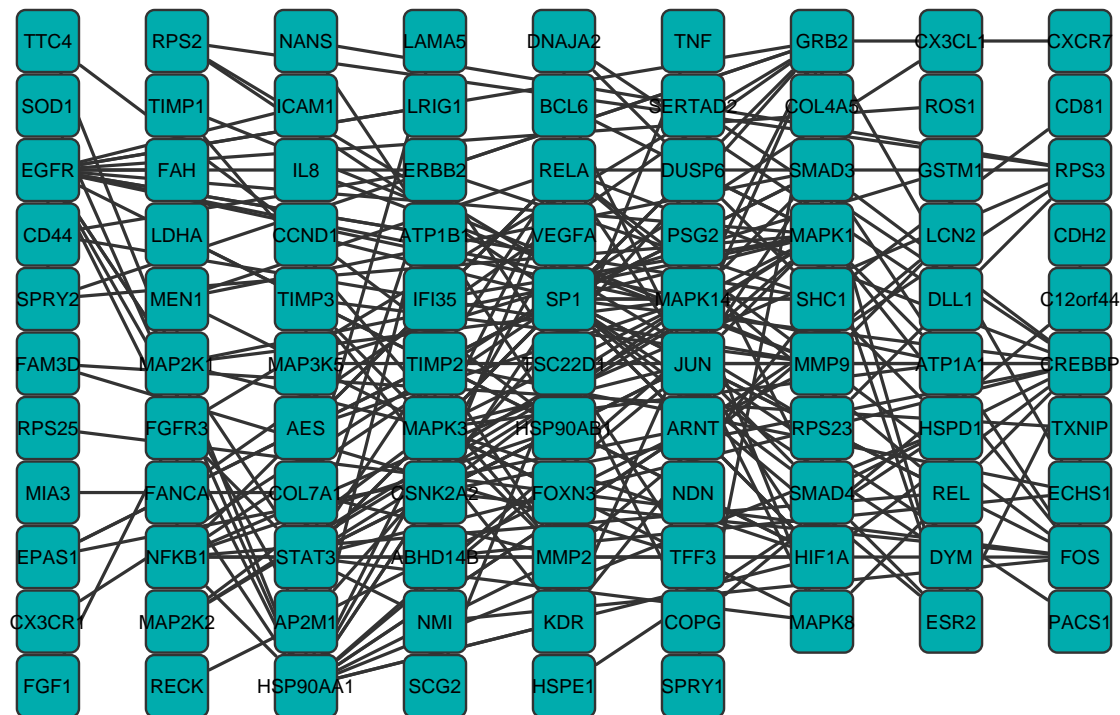

Supplement: File S6 — The pdf file of PPI network. (PDF) [file pone.0100094.s006.pdf]

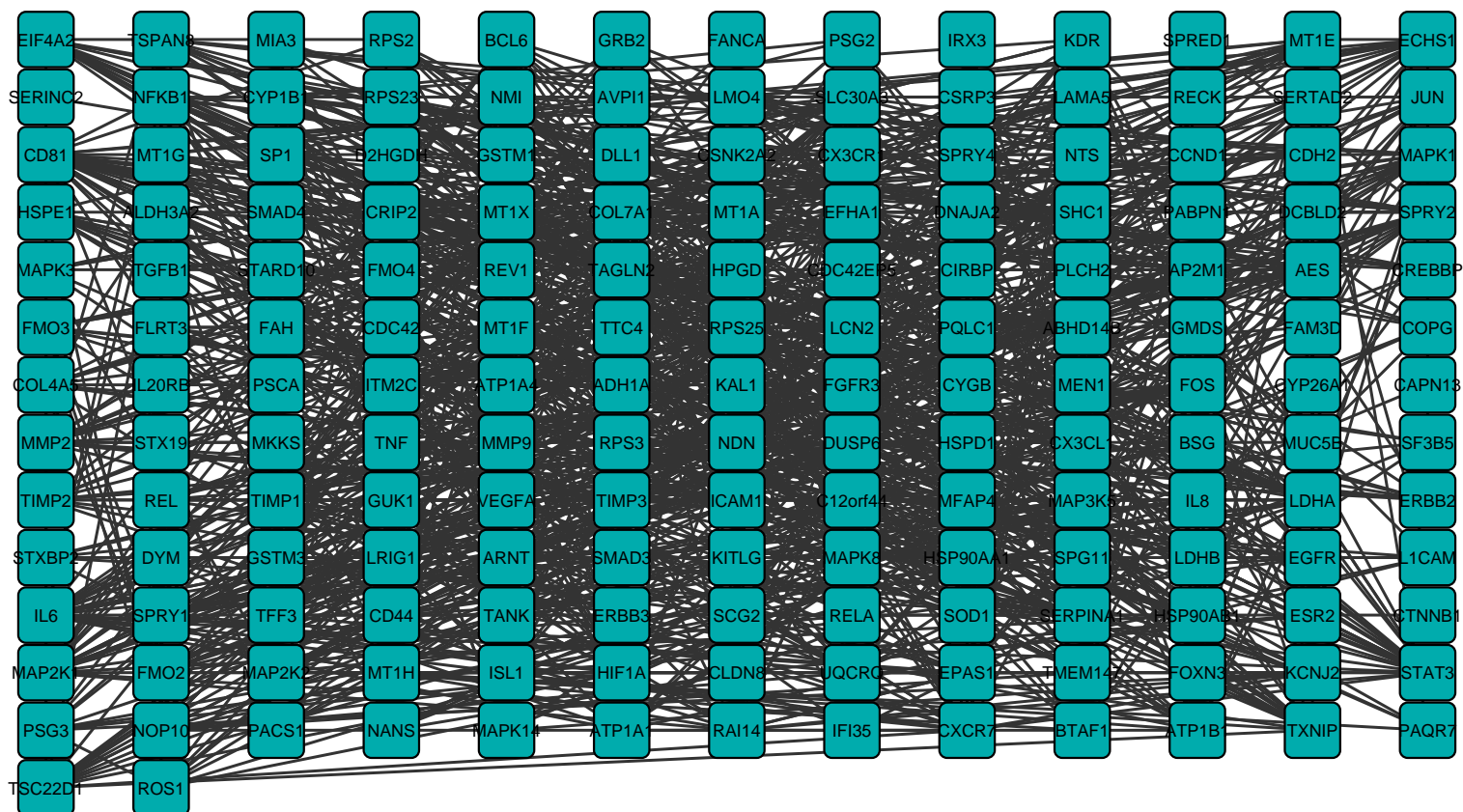

Supplement: File S7 — The pdf file of GRN network. (PDF) [file pone.0100094.s007.pdf]
